# Protective Effects of Regular Physical Activity: Differential Expression of *FGF21*, *GDF15,* and Their Receptors in Trained and Untrained Individuals

**DOI:** 10.3390/ijms26157115

**Published:** 2025-07-23

**Authors:** Paulina Małkowska, Patrycja Tomasiak, Marta Tkacz, Katarzyna Zgutka, Maciej Tarnowski, Agnieszka Maciejewska-Skrendo, Rafał Buryta, Łukasz Rosiński, Marek Sawczuk

**Affiliations:** 1 Institute of Physical Culture Sciences, University of Szczecin, 70-240 Szczecin, Poland; paulina.malkowska@usz.edu.pl (P.M.); patrycja.tomasiak@usz.edu.pl (P.T.); maciej.tarnowski@pum.edu.pl (M.T.); agnieszka.maciejewska-skrendo@awf.gda.pl (A.M.-S.); rafal.buryta@usz.edu.pl (R.B.); lukasz.rosinski@usz.edu.pl (Ł.R.); 2 Department of Physiology in Health Sciences, Faculty of Health Sciences, Pomeranian Medical University, 71-210 Szczecin, Poland; marta.tkacz@pum.edu.pl (M.T.); katarzyna.zgutka@pum.edu.pl (K.Z.); 3 Faculty of Physical Culture, Gdansk University of Physical Education and Sport, 80-336 Gdansk, Poland

**Keywords:** FGF21, GDF15, physical activity, intense exercise, cytokine response, inflammation, exercise adaptation, metabolic stress, biomarkers

## Abstract

According to the World Health Organization (WHO), a healthy lifestyle is defined as a way of living that lowers the risk of becoming seriously ill or dying prematurely. Physical activity, as a well-known contributor to overall health, plays a vital role in supporting such a lifestyle. Exercise induces complex molecular responses that mediate both acute metabolic stress and long-term physiological adaptations. FGF21 (fibroblast growth factor 21) and GDF15 (growth differentiation factor 15) are recognized as metabolic stress markers, while their receptors play critical roles in cellular signaling. However, the differential gene expression patterns of these molecules in trained and untrained individuals following exhaustive exercise remain poorly understood. This study aimed to examine the transcriptional and protein-level responses in trained and untrained individuals performed a treadmill maximal exercise test to voluntary exhaustion. Blood samples were collected at six time points (pre-exercise, immediately post-exercise, and 0.5 h, 6 h, 24 h, and 48 h post-exercise). Gene expression of *FGF21*, *GDF15*, *FGFR1* (fibroblast growth factor receptors), *FGFR3*, *FGFR4*, *KLB* (β-klotho), and *GFRAL* (glial cell line-derived neurotrophic factor receptor alpha-like) was analyzed using RT-qPCR, while plasma protein levels of FGF21 and GDF15 were quantified via ELISA. The results obtained were statistically analyzed by using Shapiro–Wilk, Mann–Whitney U, and Wilcoxon tests in Statistica 13 software. Untrained individuals demonstrated significant post-exercise upregulation of *FGFR3*, *FGFR4*, *KLB*, and *GFRAL*. FGF21 and GDF15 protein levels were consistently lower in trained individuals (*p* < 0.01), with no significant correlations between gene and protein expression. Trained individuals showed more stable expression of genes, while untrained individuals exhibited transient upregulation of genes after exercise.

## 1. Introduction

Consistent physical activity has been proven to enhance heart health, reducing the likelihood of conditions such as heart disease, stroke, and high blood pressure, among other well-documented benefits [1,2,3,4,5,6]. The body’s response to exercise can be observed across multiple levels—from molecular and cellular to tissue, organ, and systemic. The term systemic in this context refers to effects that involve the entire body as a whole, engaging multiple physiological systems such as the cardiovascular, respiratory, endocrine, immune, and nervous systems, which work together to adapt to physical demands. However, the specific outcomes depend largely on the type, duration, and intensity of the exercise regimen, highlighting the importance of tailoring workouts to individual needs for optimal results [7]. Małkowska and Sawczuk showed, in their review, that intense exercise can lead to negative effects such as a pro-inflammatory response characterized by an increase in pro-inflammatory cytokines [8]. In recent decades, significant breakthroughs have revealed that a wide range of proteins, collectively referred to as exerkines, are released during and after exercise. These molecules, including fibroblast growth factor 21 (FGF21) and growth differentiation factor 15 (GDF15), play a crucial role in regulating energy metabolism and facilitating communication between various organs and tissues, including skeletal muscle, adipose tissue, the heart, and the brain [9,10]. By influencing these interactions, exerkines contribute to the overall benefits of physical activity, helping to optimize the body’s physiological responses to exercise and improve overall health [10].

Fibroblast growth factor 21 (FGF21) is a protein recognized as a myokine, but it is also secreted by other organs, including the liver, heart, and both types of adipose tissue—white and brown (WAT and BAT) [11,12,13,14,15,16]. FGF21 is essential for the regulation of metabolism, influencing processes such as glucose uptake, lipid metabolism, and overall energy balance. It has been shown that FGF21, as an anti-inflammatory agent, suppresses various metabolic pathways, including gluconeogenesis, fatty acid oxidation, lipolysis, ketogenesis, and lipogenesis, while also affecting the growth hormone signaling pathway [17,18,19]. FGF21 levels are typically low in skeletal muscle under normal conditions, and the majority of circulating FGF21 comes from the liver [11]. However, its production increases muscle tissue as the body responds to stress conditions like starvation, mitochondrial dysfunction, endoplasmic reticulum stress, obesity, and aging [20]. This myokine is considered a marker of metabolic stress and has gained attention as a potential biomarker for muscle-specific mitochondrial disorders due to its role in muscle energy regulation and stress adaptation [21,22]. FGF21 exerts its biological effects by binding to fibroblast growth factor receptors (FGFR1 and FGFR2) in conjunction with its essential co-receptor, β-klotho (KLB). In the absence of FGF21, FGFR and KLB form a stable 1:1 heterocomplex. However, when FGF21 is present, it induces a conformational change in the FGFRs, causing dimerization. This results in the formation of a 1:2 heterocomplex with KLB, leading to downstream signaling events that initiate the cellular response [23]. This receptor activation allows FGF21 to influence tissues where both FGFR and KLB are expressed, including white adipose tissue, skeletal muscle, the heart, pancreas, and brown adipose tissue. By engaging these tissues, FGF21 regulates key processes like energy metabolism, glucose homeostasis, and lipid metabolism [24].

Growth differentiation factor 15 (GDF15), also known as macrophage inhibitory cytokine 1 (MIC-1), is a member of the transforming growth factor-β (TGF-β) superfamily of stress sensors [25]. GDF15 plays a role in regulating energy metabolism and appetite, potentially by influencing mitochondrial functions such as biogenesis, thermogenesis, and fatty acid breakdown [26]. The discovery of its receptor, the glial cell line-derived neurotrophic factor receptor alpha-like (GFRAL) in the hindbrain, has highlighted its anorectic effects, linking GDF15 to appetite suppression [27]. However, the precise role of GDF15 in health and disease remains debatable. Elevated levels of GDF15 have been observed in a variety of conditions, including cardiovascular diseases, insulin resistance, type 2 diabetes, neurodegenerative disorders, chronic kidney disease, and various cancers. Despite these associations with disease progression, GDF15 is also thought to have protective effects, helping to mitigate the damage caused by stress and injury [28]. GDF15 exhibits both anti-inflammatory and pro-inflammatory effects, depending on the context and the specific disease. In inflammation-related conditions, it can act as a modulator of the immune response, either suppressing or promoting inflammation, which suggests its dual role in regulating the body’s inflammatory processes [29]. Research in animal models suggests that GDF15 may promote muscle cell apoptosis [30,31], but paradoxically, the absence of GDF15 results in an enhanced skeletal muscle stress response after exercise, with a stronger activation of stress markers such as Atf3, Atf6, and Xbp1s [32]. In humans, GDF15 levels are notably elevated in individuals with sarcopenia or muscle wasting compared to age-matched healthy individuals. Studies have also shown that GDF15 levels are inversely correlated with muscle mass, strength (such as hand-grip strength), and muscle cross-sectional area, indicating that GDF15 may play a role in muscle tissue loss [33].

Although expression of FGF21 and GDF15 has been studied, significant gaps in research remain. To address this, we aimed to investigate the impact of intense exercise, specifically through a progressive refusal test, on the expression of these proteins and their corresponding genes. To gain deeper insights into the underlying mechanisms, we also examined the expression levels of the receptors for both FGF21 and GDF15. Additionally, recognizing that exercise effects may vary depend on fitness level, we included two groups of participants in our study: professional athletes and untrained individuals. This study allowed us to explore potential differences in response to exercise based on training status.

## 2. Results

### 2.1. Comparison Gene Expression Between Trained and Non-Trained Group

**Figure 1:**
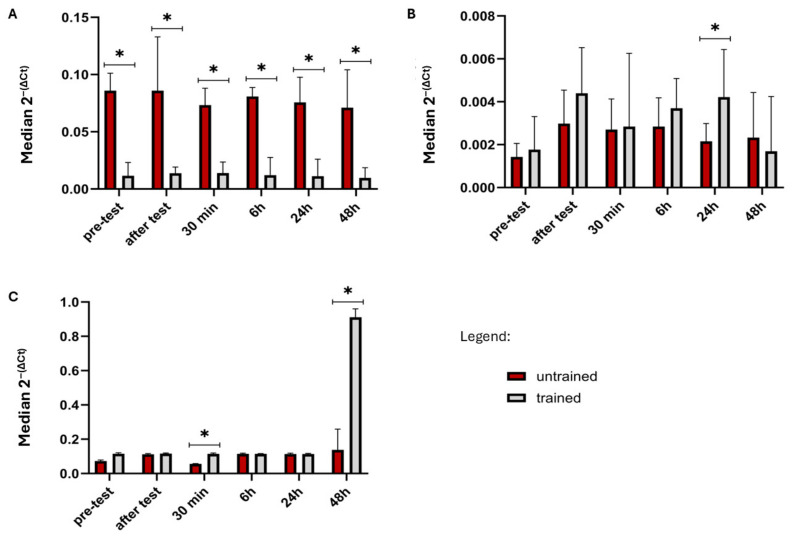
(**A**) *FGFR1* expression level changes in trained and untrained groups between pre-test and five post-tests after physical activity. (**B**) *FGFR3* expression level changes in trained and untrained groups between pre-test and five post-tests after physical activity. (**C**) *FGFR4* expression level changes in trained and untrained groups between pre-test and five post-tests after physical activity. The graph substitutes the median value of 2^−(∆CT)^ obtained from real-time PCR with interquartile range. * Significant differences between groups (*p* < 0.05).

*FGF21*, *GDF15*, *KLB,* and *GFRAL* did not show significant differences between groups (*p* > 0.05, Table 1) in any time point. Only *FGFR1* expression exhibited a statistically significant difference between the two groups during every time point. During pre-test *FGFR1* expression was significantly higher in the untrained group than in the trained group (*p* < 0.05). This result was maintained during all measurements (Table 1, Figure 1A).

*FGFR3* expression was significantly lower in untrained individuals (*p* < 0.05) only at 24 h after test (Table 1, Figure 1B). No significant changes were observed between groups at the other time points.

*FGFR4* expression showed a statistically significant difference between the groups (*p* < 0.05) 30 min after test (Table 1, Figure 1C), where *FGFR4* was significantly upregulated in the trained group. At the next time point, 6 h after exercise, this effect was no longer present (*p* < 0.05). *FGFR4* gene showed significantly higher *(p* < 0.05, Figure 1C*)* expression also 24 h after the test in the trained group versus the untrained group.

### 2.2. Comparison Gene Expression Between Time Points in Trained and Non-Trained Group

Table 2 shows the results obtained by comparing the expression changes between the different time points in the trained and untrained individuals.

In the untrained group there were no statistically significant differences in *FGF21*, *GDF15,* and *FGFR1* expression at any time point as compared to baseline (*p* > 0.05 for all comparisons). In the trained group the observation was similar. There was no statistically significant difference in any post-exercise time point in *FGF21*, *GDF15*, *FGFR1*, *FGFR3*, *KLB,* and *GFRAL* expression as compared to baseline (*p* > 0.05 in all comparisons).

**Figure 2:**
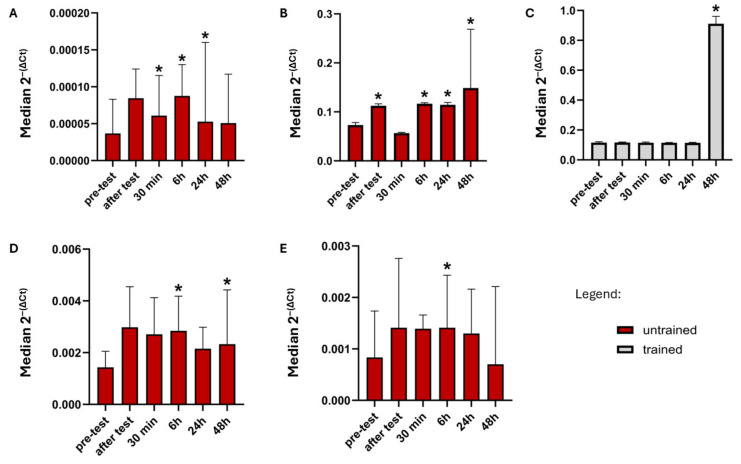
(**A**) FGFR3 expression level changes in untrained group between pre-test and five post-tests after physical activity. (**B**) FGFR4 expression level changes in untrained group between pre-test and five post-tests after physical activity. (**C**) FGFR4 expression level changes in trained group between pre-test and five post-tests after physical activity. (**D**) KLB expression level changes in untrained group between pre-test and five post-tests after physical activity. (**E**) GFRAL expression level changes in untrained group between pre-test and five post-tests after physical activity. The graph substitutes the median value of 2^−(∆CT)^ obtained from real-time PCR with interquartile range. * Significant difference between this time point and resting level (*p* ≤ 0.05).

Statistically significant differences in *FGFR3* expression were observed in the untrained group at two time points compared to baseline. The first was a significant increase in *FGFR3* expression (*Z =* 2.131, *p* < 0.05, Figure 2A) at 6 h post-exercise. The second was a significant increase in *FGFR3* expression (*p* < 0.05, *p =* 0.0464, Figure 2A) at 48 h post-exercise. No statistically significant changes were observed at other time points.

*FGFR4* expression increased significantly at multiple time points in the untrained group. Significant increases compared to baseline was observed immediately post-exercise (*Z* = 2.411, *p* < 0.05, Figure 2B), at 6 h post-exercise (*Z* = 2.691, *p* = 0.0071, Figure 2B), 24 h post-exercise (*Z* = 2.481, *p* < 0.05, Figure 2B), and 4 h post-exercise (*Z =* 2.760, *p* < 0.05, Figure 2B). A significant increase was also observed in the trained group 48 h after the test (*Z* = 3.408, *p* < 0.05, Figure 2C).

The results in the untrained group suggest that *KLB* expression significantly increased between 0.5 h (*Z* = 2.510, *p* < 0.05, Figure 2D) and pre-test. Expression remained elevated, with statistical significance (*Z =* 2.6207, *p* < 0.05, Figure 2D) at 6 h after test and at 24 h post-exercise (*Z* = 1.992, *p* < 0.05, Figure 2D).

*GFRAL* expression significantly increased at 6 h post-exercise (*Z* = 2.341, *p* < 0.05, Figure 2E) in untrained group. In contrast to the untrained group, *GFRAL* expression remained stable across all time points in trained individuals. There were no statistically significant differences across all comparisons (*p* > 0.05).

### 2.3. Analysis of ELISA Results

The Mann–Whitney U test results show a statistically significant difference in FGF21 and GDF15 protein concentrations between the trained and untrained groups across all six time points. The *p*-values are well below the conventional significance threshold of 0.05.

**Figure 3:**
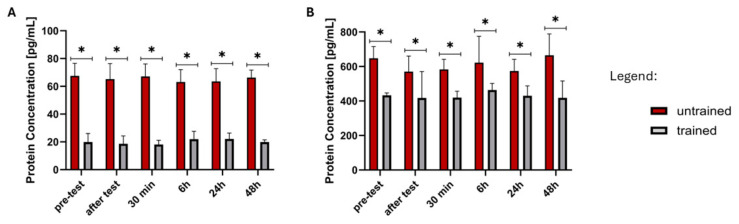
Visualization of FGF21 (**A**) and GDF15 (**B**) proteins expression that showed significant changes between untrained and trained groups over all time points. * Significant difference between groups (*p* ≤ 0.05).

The sum of ranks for the trained group is consistently lower than for the untrained group, which suggests that FGF21 concentrations were systematically lower in the trained group across all time points (Figure 3A). The U-values are at their minimum (0 or close to 0 in most comparisons), and the Z-scores exceed 4 in absolute magnitude for all comparisons. This suggests a strong effect size, reinforcing the robustness of the difference. The statistical significance persists across all time points (pre-test, after test, 0.5 h, 6 h, 24 h, 48 h), indicating a stable and systematic difference in FGF21 levels between groups rather than a transient effect of exercise or recovery.

The results for GDF15 also showed significant differences between the trained and untrained groups. All six time points show *p*-values below 0.05, with most comparisons demonstrating highly significant differences (*p* < 0.01, Figure 3B). This suggests that the concentration of GDF15 differs consistently between the two groups. The sum of ranks for the untrained group is higher than that of the trained group in most cases, indicating that GDF15 concentrations were generally higher in untrained individuals across multiple time points. The largest difference appears at time point 1 (pre-test) with a highly significant *p*-value (*p* < 0.0001), suggesting a baseline disparity in GDF15 levels between trained and untrained individuals. Although differences remain statistically significant at later time points, the variation in U-values suggests some temporal fluctuations in GDF15 concentrations.

The analysis examines also differences in FGF21 and GDF15 expression between time points separately for the trained and untrained groups.

**Figure 4:**
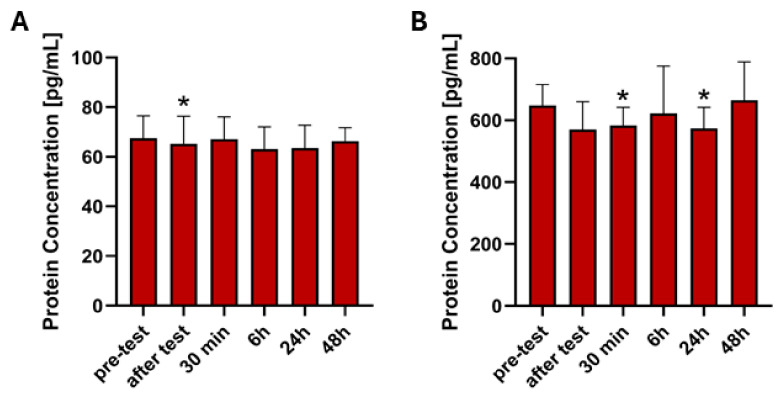
Visualization of FGF21 (**A**) and GDF15 (**B**) protein expression that showed significant changes in untrained group between pre-test and five post-tests after physical activity. * Significant difference between this time point and resting level (*p* < 0.05).

A significant decrease in FGF21 expression was observed immediately after the test in the untrained group compared to the pre-test level (*Z* = 2.06, *p* < 0.05, Table 3, Figure 4A), suggesting an acute response to exercise. No statistically significant changes were observed at 30 min, 6 h, 24 h, or 48 h post-exercise compared to the pre-test, indicating a return to baseline levels after the initial response. In the trained group there were no statistically significant differences observed between any time points.

A statistically significant decrease in GDF15 expression in untrained group was observed 30 min post-exercise (*Z* = 2.48, *p* < 0.05, Table 3, Figure 4B), and a significant decrease was noted at 24 h compared to baseline (*Z* = 1.99, *p* < 0.05, Table 3, Figure 4B). No statistically significant changes were observed at after test, 6 h, or 48 h post-exercise compared to the pre-test. In the trained group, no statistically significant differences in GDF15 expression were observed between any time points.

### 2.4. Correlation Analysis

A correlation analysis was also performed between the protein expression of FGF21 and GDF15 and the expression of the genes encoding them.

In the untrained group, across all six time points, the correlation coefficients (R Spearman) remain low, ranging from −0.264 to 0.326. None of the correlations reach statistical significance (*p* > 0.05 for all comparisons), indicating that ELISA and PCR measurements do not show a strong or consistent relationship in this group. Similar to the untrained group, the correlation coefficients are weak and inconsistent, ranging from −0.111 to 0.239 in the trained group. None of the time points show a significant correlation (*p* > 0.05 for all comparisons).

In the untrained group, all correlation coefficients are negative, suggesting an inverse or weak relationship between GDF15 gene expression and protein levels. In contrast to the untrained group, the trained group demonstrated mostly positive correlations, but no correlations reached statistical significance.

The results of the correlation are shown in Table 4.

## 3. Discussion

Our study investigated the differential expression of genes related to metabolic and inflammatory responses (*FGF21*, *GDF15*, *FGFR1*, *FGFR3*, *FGFR4*, *KLB*, and *GFRAL*) and the corresponding protein levels of FGF21 and GDF15 in trained and untrained individuals following exhaustive exercise. The results provide novel insights into the molecular adaptations associated with chronic physical training and the acute response to exercise stress.

Among the analyzed genes, *FGFR1* consistently exhibited significantly higher expression levels in trained individuals compared to their untrained counterparts across all time points. This suggests that regular physical training enhances the basal and exercise-induced expression of FGFR1, potentially contributing to improved cellular signaling related to energy metabolism and tissue repair. Previous studies have suggested that FGFR1 plays a central role in mediating the metabolic effects of FGF21, including enhanced glucose uptake and lipid oxidation [34]. However, we did not observe changes in *FGFR1* gene expression after the exercise as compared to resting values in either the trained or untrained group. A study involving patients selected after coronary artery bypass grafting showed that aerobic and resistance training did not significantly affect changes in FGFR1 protein levels when comparing resting values to values immediately after exercise [35]. However, the same study showed that a group of patients who underwent an 8-week training program had significantly higher levels of FGFR1 expression compared to a control group that did not participate in training [35]. These observations are in line with our results, suggesting that FGFR1 can be used as a potential marker of the physical fitness level.

*FGFR4* expression showed a significant difference between groups at 0.5 h post-exercise, with higher expression observed in untrained individuals. However, this difference did not persist at later time points. These findings indicate also that in untrained individuals, *FGFR4* expression is significantly upregulated immediately after exercise and remains enhanced for at least 48 h. This prolonged response may be associated with muscle repair and adaptation processes following acute physical exertion. This may indicate a transient exercise-induced response. FGFR4 is involved in skeletal muscle differentiation and tissue repair [36]. This delayed response may reflect a more efficient regulation of FGFR4 in trained individuals, potentially due to enhanced muscle repair mechanisms that require longer activation periods. To our knowledge, this is the first study to evaluate the effect of exercise on *FGFR4* gene expression in trained and untrained individuals.

Our results show a significantly lower expression of *FGFR3* at 24 h after exercise in untrained individuals compared to trained individuals. A significant increase in expression was observed 6 h after exercise and 24 h after the test in the untrained group. To our knowledge, the effect of exercise on FGFR3 and the understanding of the role and potential of FGFR3 in metabolic stress and muscle regeneration is currently a research gap. Based on the available studies, it has been established that a key role of FGFR3 is to regulate osteogenesis and bone remodeling by affecting osteoblasts and growth plates [37]. Studies in mice have shown that conditional inactivation of FGFR3 in chondrocytes leads to excessive osteogenesis, increased osteoblast numbers, and enhanced bone remodeling [37,38]. This suggests that FGFR3 is a key regulator of bone cell proliferation and differentiation and the dynamic balance between bone resorption and bone formation. Although FGFR3 is best known for regulating cartilage and bone growth, its effect on muscle tissue is not well understood. However, other FGFR receptors (e.g., FGFR1 and FGFR4) have key functions in muscle regeneration and metabolism [34,36]. As FGFR3 is part of the same receptor family, it can be assumed that it is also involved in pathways regulating muscle adaptation to exercise and regenerative processes. In our study, *FGFR3* expression significantly increased 24 h after exercise in the trained group. This may suggest that its levels are regulated by training intensity and regenerative processes.

In the untrained group, significant changes in *KLB* gene expression were observed at multiple time points relative to the pre-test value. *KLB* gene expression in the untrained group is significantly altered post-exercise, with the most notable changes occurring at 6, 24, and 48 h after the test, likely reflecting the physiological stress and recovery processes associated with an intense physical challenge. The lack of significant findings in the trained group may suggest that regular exercise leads to a more stable regulation of *KLB* gene expression. This could be indicative of an adaptive response to training, where the impact of acute exercise on *KLB* gene expression is less pronounced in trained individuals compared to untrained ones.

We noted no significant differences in *GFRAL* gene expression between the study groups, although there was a moderate trend towards higher expression in the trained subjects. However, when considering the differences in gene expression after the performance test compared to resting values, we noted a significant increase in the untrained group at 6 and 48 h, where the increase was greatest. GDF15 has been identified as a stress-responsive cytokine with a potential role in energy metabolism and mitochondrial adaptation [39]. The occurrence of the greatest increase in the expression of *GFRAL*, the GDF15 receptor, suggests that the lack of regular physical activity may influence the body’s less effective adaptation to exercise-induced stress. The results suggest that non-exercisers have a poorer ability to respond to physical stress, which may lead to adverse changes in energy metabolism and mitochondrial function.

It has been reported that in male marathon runners, plasma levels of GDF15 and FGF21 increased significantly immediately following the race, with levels returning to baseline after 48 h [40]. In contrast, a 5-week endurance training program in elderly men, consisting of three sessions per week on a cycle ergometer, led to a decrease in serum FGF21 levels [41]. Interestingly, GDF15 levels seem to increase in response to various types of physical activity. In healthy men, GDF15 levels significantly increased during exercise and two hours post-exercise [42]. Moderately trained men showed an increase in response to both endurance and resistance exercise, while triathletes exhibited a significantly increase after 4 h of cycling, with levels returning to baseline within 24 h [43]. Surprisingly, in our study, neither *FGF21* nor *GDF15* gene expression showed significant differences between trained and untrained individuals at any time point. Several factors might explain this discrepancy. First, the exercise protocol used in our study may differ in intensity or duration from those employed in the studies mentioned, potentially influencing the response of these markers. Moreover, the type of exercise, the physical fitness of the participants, and individual variability in metabolic responses could account for the differences in outcomes. Given that FGF21 is widely regarded as an exercise-responsive factor [44], the absence of significant changes may be attributed to inter-individual variability, tissue-specific expression patterns, or the specific characteristics of our study population. Similarly, while GDF15 has been proposed as an exercise-induced myokine [27], our findings suggest that its transcriptional regulation in blood may be minimal or delayed beyond the 48 h window assessed in this study. These considerations highlight the need for further studies that carefully control for these variables to better understand the relationship between physical activity and the regulation of FGF21 and GDF15.

ELISA analysis revealed that both FGF21 and GDF15 protein levels were significantly lower in trained individuals compared to the untrained group across all time points. This consistent difference suggests that chronic exercise training suppresses the systemic levels of these proteins, which are often considered markers of metabolic stress and mitochondrial dysfunction. The significantly higher levels in untrained individuals may indicate a greater metabolic strain in response to exercise, further highlighting the protective effect of regular physical activity. According to the information available to us, there is currently no work comparing protein concentration levels in trained and untrained individuals in response to intense exercise.

Interestingly, despite the observed group differences in protein levels, correlation analysis between gene and protein expression revealed weak and inconsistent relationships. Neither trained nor untrained individuals demonstrated significant correlations between mRNA and protein levels for FGF21 and GDF15, suggesting that post-transcriptional and post-translational mechanisms play a critical role in regulating these proteins. This finding aligns with previous reports indicating that FGF21 and GDF15 expression is subject to complex regulatory processes, including hormonal signaling, tissue-specific secretion, and degradation dynamics.

An important next step in expanding our understanding of exercise-induced molecular adaptations would be to investigate the role of brain-derived neurotrophic factor (BDNF), a neurotrophin increasingly recognized for its involvement in both metabolic regulation and exercise-mediated plasticity. BDNF expression has been shown to increase in response to acute and chronic physical activity, particularly in skeletal muscle and the central nervous system, where it promotes mitochondrial biogenesis, fatty acid oxidation, and glucose metabolism through AMPK and PGC-1α pathways [45]. In humans, both acute aerobic and chronic training interventions have been shown to elevate peripheral BDNF levels, with aerobic exercise increasing resting concentrations [46]. Additionally, evidence suggests that BDNF may exert anti-inflammatory effects and influence systemic responses to physical stress, making it a promising biomarker for evaluating exercise-induced adaptations alongside FGF21 and GDF15 [46,47]. Future studies should therefore consider including BDNF measurements—both at the gene and protein levels—to provide a more integrated view of how neurotrophic and metabolic pathways coordinate the body’s response to exercise stress.

### Limitations

While our study provides valuable insights into the differential gene expression and protein levels of metabolic and inflammatory markers in trained and untrained individuals following exhaustive exercise, several limitations should be acknowledged.

Firstly, the relatively small sample size may limit the generalizability of our findings. Although we observed significant differences in gene expression and protein levels between groups, a larger cohort would enhance statistical power and allow for more robust conclusions. Future studies with expanded sample sizes could confirm the observed trends and minimize inter-individual variability.

Our study focused exclusively on peripheral blood samples, which may not fully reflect transcriptional changes occurring within skeletal muscle or other metabolically active tissues. Given that FGF21, GDF15, and FGFRs play crucial roles in tissue-specific metabolic regulation, further studies should be conducted in an animal model and include multi-tissue analyses that would provide a more comprehensive understanding of molecular adaptations to exercise.

Another limitation is the lack of direct assessment of protein expression at the tissue level. While we measured systemic protein concentrations of FGF21 and GDF15 using ELISA, local protein expression within muscle or liver tissues may differ significantly due to post-transcriptional regulation. Future animal model studies using Western blotting, immunohistochemistry, or proteomic approaches in relevant tissues would provide deeper mechanistic insights.

Additionally, the study did not control for dietary intake, which could have influenced both gene expression and protein levels. Nutritional status, macronutrient composition, and caloric intake can significantly modulate metabolic responses to exercise. Standardizing dietary intake in future studies would help minimize confounding effects and provide more accurate interpretations of exercise-induced molecular changes.

Despite these limitations, our findings contribute to the growing body of literature on exercise-induced molecular adaptations and provide a foundation for future research investigating the role of metabolic and inflammatory markers in training status and exercise recovery.

## 4. Materials and Methods

### 4.1. Participants

The entire experimental protocol was approved by the Bioethics Committee at the University of Szczecin on 22 May 2024 (approval number: US.003.5.2024). All participants were provided with an informed consent form and a detailed information sheet outlining the study’s purpose, procedures, potential risks, and benefits of participation. They were given ample time to read both documents and were encouraged to ask questions if any details were unclear. After ensuring that they fully understood the information provided, each participant signed the informed consent form, agreeing to take part in the study. They were also reassured that the study would be conducted anonymously and that their results would remain confidential. Fifteen football players (n = 15) aged 20–25 were recruited for the study in the “trained” group (T). Inclusion criteria for the “trained” group were male gender, documented history of systematic sports training for more than 10 years (average of 12 training hours/week), good health (no concurrent injuries), negative medical history for cardiovascular conditions, central nervous system, mental disorders, head injuries and other diseases that could directly affect the study, not taking dietary supplements or medications during the study that could directly affect the results. Fifteen participants (n = 15) aged 20–25 were recruited for the study in the “untrained” group (UT). Inclusion criteria for the “untrained” group were male gender, similarity with the study group in terms of age and characteristics of general morphological indices, low level of physical activity (self-reported by each participant using the Global Physical Activity Questionnaire, GPAQ), good health (no comorbid injuries), negative medical questioning for cardiovascular disorders, central nervous system disorders, mental disorders, head injuries and other diseases that could directly affect the experiment, not taking dietary supplements or medications during the study that could directly affect the results.

### 4.2. Physical Activity

A treadmill maximal voluntary exhaustion protocol was performed by each subject. The test was designed to determine individual exercise tolerance and provoke maximal metabolic and cardiovascular stress. Participants began the test with a 3-min warm-up at a self-selected comfortable walking pace, followed by a progressive increase in running speed and/or incline every 2 min until volitional exhaustion was reached. Exhaustion was defined as the point at which the participant could no longer maintain the required speed despite verbal encouragement and demonstrated clear signs of fatigue (e.g., inability to continue running). The protocol was adapted to individual fitness levels to ensure comparability of physiological load across trained and untrained participants. Heart rate and perceived exertion were continuously monitored, and the test was immediately terminated upon reaching voluntary maximal effort or safety-related criteria.

### 4.3. Sample Collection

Venous blood samples were collected from study participants before (pre-test), immediately after the exercise (0 h), and 30 min (0.5 h), 6 h (6 h), 24 h (24 h), and 48 h (48 h) after the test into tubes with stabilizer (RNAlater). In addition, blood plasma was collected for evaluation of protein levels by enzyme-linked immunosorbent assay (ELISA).

### 4.4. Isolation of the RNA and cDNA Synthesis

Total RNA was extracted from blood samples using the GeneMATRIX Universal RNA Purification Kit (EurX, Gdańsk, Poland), designed for RNA isolation, according to the manufacturer’s protocol. The resulting isolates tested spectrophotometrically for RNA quantity and quality were reverse-transcribed (RT) using OneStep RT-PCR Kit (EurX, Gdańsk, Poland) via the polymerase chain reaction (RT-PCR) technique, and the resulting cDNA samples were stored at −20 °C for further molecular analysis.

### 4.5. Real-Time PCR

To assess the expression of the *FGF21*, *GDF15*, *FGFR1*, *FGFR3*, *FGFR4*, *GFRAL* and *KLB* genes (Table 5), quantitative real-time PCR (RT-qPCR) was conducted in duplicates of each sample using the StepOne real-time PCR system (Applied Biosystems, Foster City, CA, USA) and T100 thermal cycler (Bio-Rad, Hercules, CA, USA). Fluorescence detection during the RT-qPCR process was performed in 96-well low-skirted plates (Applied Biosystems, Foster City, CA, USA) with the SsoAdvanced Universal SYBR Green SuperMix (Bio-Rad, Hercules, CA, USA) according to the manufacturer’s protocol. The amplification conditions were as follows: an initial denaturation at 95 °C for 30 s, followed by 40 cycles of denaturation at 95 °C for 10 s and annealing/extension at 60 °C for 30 s, with plate reading occurring at the end of each cycle. To normalize the data, the mRNA expression of the housekeeping gene coding for human glyceraldehyde 3-phosphate dehydrogenase (GAPDH) was used as a reference.

### 4.6. ELISA

Blood plasma was collected from all study participants at the indicated time points. Plasma levels of FGF21 (SEC918Hu) and GDF15 (SEC034Hu) were determined using an enzyme immunoassay (Cloud-Clone Corp., Katy, TX, USA) according to the manufacturer’s instructions.

### 4.7. Statistical Analysis

The expression results of individual genes were obtained by calculating the 2^−ΔCt^ value, which was then subjected to statistical analysis. For statistical analysis, tests of statistical significance appropriate to the characteristics of the data were applied. In the first step, the normality of the distribution of the variables was assessed using the Shapiro–Wilk test. Most of the distributions were significantly different from normal, so non-parametric tests were used to compare groups: Mann–Whitney U for independent samples and Wilcoxon paired rank order test for dependent samples.

Statistical analyses were performed using Statistica 13 software. A *p* value < 0.05 was considered statistically significant. Due to the lack of a normal distribution, medians were used to visualize the data. The software in which the figures were prepared was GraphPad Prism 10.

## 5. Conclusions

In conclusion, our study provides new insights into the molecular adaptations of trained and untrained individuals in response to exhaustive exercise. We found significant differences in the expression of genes involved in metabolic and inflammatory pathways, such as *FGFR1*, *FGFR3*, *FGFR4*, and *KLB*, with trained individuals showing a more stable and regulated gene expression profile. Notably, *FGFR1* expression was consistently higher in trained individuals, suggesting its potential as a marker of training status. Conversely, *FGFR4* exhibited a transient downregulation in untrained individuals, indicating a delayed response to exercise-induced stress. While no significant changes were observed in *FGF21* and *GDF15* gene expression, protein levels of these markers were lower in trained individuals, further emphasizing the protective effects of regular physical activity. These findings underscore the complex regulatory mechanisms underlying exercise-induced adaptations and highlight the need for further research to explore the interplay between gene expression, protein levels, and physical activity.

## Figures and Tables

**Table 1:** Summary table of gene expression differences between trained and untrained group across six time points.

Gene	Pre-Exercise	Immediately Post-Exercise	0.5 h Post- Exercise	6 h Post- Exercise	24 h Post- Exercise	48 h Post- Exercise
*FGF21*	UT < T (Z = −1.198, *p* = 0.231)	UT < T (Z = −0.415, *p* = 0.678)	UT < T (Z = −0.967, *p* = 0.333)	UT < T (Z = −1.267, *p* = 0.205)	UT < T (Z = −0.760, *p* = 0.447)	UT < T (Z = −0.184, *p* = 0.854)
*GDF15*	UT < T (Z = −1.059, *p* = 0.289)	UT < T (Z = −0.829, *p* = 0.407)	UT < T (Z = −1.152, *p* = 0.249)	UT < T (Z = −1.013, *p* = 0.311)	UT < T (Z = −1.106, *p* = 0.269)	UT < T (Z = −0.553, *p* = 0.580)
*FGFR1*	UT > T (Z = 3.869, ***p* = 0.0001**)	UT > T (Z = 3.824, ***p* = 0.0001**)	UT > T (Z = 3.869, ***p* = 0.0001**)	UT > T (Z = 3.271, ***p* = 0.0011**)	UT > T (Z = 3.685, ***p* = 0.0002**)	UT > T (Z = 3.962, ***p* = 0.00007**)
*FGFR3*	UT < T (Z = −1.013, *p* = 0.311)	UT < T (Z = −1.289, *p* = 0.197)	UT < T (Z = −1.013, *p* = 0.311)	UT < T (Z = −0.829, *p* = 0.407)	UT < T (Z = −2.257, ***p* = 0.024**)	UT > T (Z = 0.253, *p* = 0.800)
*FGFR4*	UT < T (Z = −1.843, *p* = 0.065)	UT < T (Z = −0.576, *p* = 0.565)	UT < T (Z = −2.303, ***p* = 0.021**)	UT < T (Z = 0.345, *p* = 0.730)	UT < T (Z = −1.681, *p* = 0.093)	UT < T (Z = −2.879, ***p* = 0.004**)
*GFRAL*	UT < T (Z = −1.474, *p* = 0.140)	UT < T (Z = −0.875, *p* = 0.381)	UT < T (Z = −1.036, *p* = 0.300)	UT < T (Z = −1.566, *p* = 0.117)	UT < T (Z = −1.152, *p* = 0.249)	UT < T (Z = 1.244, *p* = 0.214)
*KLB*	UT < T (Z = −0.921, *p* = 0.357)	UT < T (Z = −0.553, *p* = 0.580)	UT < T (Z = −0.645, *p* = 0.519)	UT < T (Z = −1.129, *p* = 0.259)	UT < T (Z = −0.345, *p* = 0.730)	UT < T (Z = −0.184, *p* = 0.854)

UT—untrained group; T—trained group; Z—result of the test of differences for two independent groups; significance level *p* < 0.05 in bold.

**Table 2:** Summary table that consolidates the results of the statistical analysis for all genes, highlighting significant differences between time points and resting level (*p* < 0.05).

Gene	Group	After Test	0.5 h	6 h	24 h	48 h
*FGF21*	UT	nd	nd	nd	nd	nd
	T	nd	nd	nd	nd	nd
*GDF15*	UT	nd	nd	nd	nd	nd
	T	nd	nd	nd	nd	nd
*FGFR1*	UT	nd	nd	nd	nd	nd
	T	nd	nd	nd	nd	nd
*FGFR3*	UT	nd	nd	**↑ (Z = 2.132,** ***p* = 0.0330)**	nd	**↑ (Z = 1.992,** ***p* = 0.0464)**
	T	nd	nd	nd	nd	nd
*FGFR4*	UT	**↑ (Z = 2.411,** ***p* = 0.0159)**	nd	**↑ (Z = 2.691,** ***p* = 0.0071)**	**↑ (Z = 2.481,** ***p* = 0.0131)**	**↑ (Z = 2.760,** ***p* = 0.0058)**
	T	nd	nd	nd	nd	**↑ (Z = 3.408,** ***p* = 0.0007)**
*KLB*	UT	nd	**↑ (Z = 2.510,** ***p* = 0.0121)**	**↑ (Z = 2.621,** ***p* = 0.0088)**	**↑ (Z = 1.992,** ***p* = 0.0464)**	nd
	T	nd	nd	nd	nd	nd
*GFRAL*	UT	nd	nd	**↑ (Z = 2.341,** ***p* = 0.0192)**	nd	nd
	T	nd	nd	nd	nd	nd

UT—untrained group; T—trained group; nd—no statistically significant differences; Z—result of the test of differences for two independent groups; significance level *p* < 0.05 in bold; ↑—increased expression.

**Table 3:** Summary table that consolidates the results of the statistical analysis for FGF21 and GDF15 proteins, highlighting significant differences (*p* ≤ 0.05) across time points and resting level.

Protein	Group	After Test	0.5 h	6 h	24 h	48 h
FGF21	UT	**↓ (*Z =* 2.062,** ***p* = 0.0392**)	nd	nd	nd	nd
	T	nd	nd	nd	nd	nd
GDF15	UT	nd	**↓ (*Z* = 2.481,** ***p* = 0.0131**)	nd	**↓ (*Z* = 1.992,** ***p* = 0.0464**)	nd
	T	nd	nd	nd	nd	nd

UT—untrained group; T—trained group; nd—no statistically significant differences; Z—result of the test of differences for two independent groups; significance level *p* ≤ 0.05 in bold; ↓—decreased expression.

**Table 4:** A correlation analysis between the FGF21 and GDF15 genetic expression and protein level across time points.

Time Point	Gene/Protein	Spearman’s R (Untrained)	*p*-Value (Untrained)	Spearman’s R (Trained)	*p*-Value (Trained)
Pre-test	FGF21	0.0604	0.8445	−0.0268	0.9244
	GDF15	−0.0604	0.8445	0.2250	0.4201
After test	FGF21	−0.2641	0.3833	0.2390	0.3909
	GDF15	−0.2473	0.4154	0.2750	0.3212
0.5 h	FGF21	0.0385	0.9006	0.0947	0.7370
	GDF15	−0.4505	0.1223	0.3000	0.2773
6 h	FGF21	0.2393	0.4310	−0.1107	0.6945
	GDF15	−0.1099	0.7208	0.3643	0.1819
24 h	FGF21	0.3269	0.2757	0.2182	0.4346
	GDF15	−0.2143	0.4821	0.4701	0.0770
48 h	FGF21	0.0934	0.7615	0.0206	0.9419
	GDF15	−0.2967	0.3249	0.0876	0.7563

**Table 5:** Primer sequences used in the study.

Gene	Name	Forward	Reverse
*hGAPDH*	glyceraldehyde 3-phosphate dehydrogenase	TGCACCACCAACTGCTTAGC	GGCATGGACTGTGGTCATGAG
*hFGF21*	fibroblast growth factor 21	CTGCAGCTGAAAGCCTTGAAGC	GTATCCGTCCTCAAGAAGCAGC
*hFGFR1*	fibroblast growth factor receptor 1	TAATGGACTCTGTGGTGCCCTC	ATGTGTGGTTGATGCTGCCG
*hFGFR3*	fibroblast growth factor receptor 3	TCCATCTCCTGGCTGAAGAACG	TGTTCTCCACGACGCAGGTGTA
*hFGFR4*	fibroblast growth factor receptor 4	AACACCGTCAAGTTCCGCTGTC	CATCACGAGACTCCAGTGCTGA
*hKLB*	β-Klotho	AAGAGTCCACGCCAGATGTGCA	CCACACGTACAGATGAGGATCG
*hGDF15*	growth differentiation factor 15	CAACCAGAGCTGGGAAGATTCG	CCCGAGAGATACGCAGGTGCA
*hGFRAL*	glial cell-derived neurotrophic factor (GDNF) family receptor α-like	GGAGAGTAATGGAAGATGCCTGC	GAAGTCATCAGTGCAAAGACACTC

## Data Availability

Data available on request due to restrictions (e.g., privacy, legal or ethical reasons).

## Abbreviations

The following abbreviations are used in this manuscript:

FGF21	fibroblast growth factor 21
GDF15	growth differentiation factor 15
FGFR	fibroblast growth factor receptors
KLB	β-klotho
MIC-1	macrophage inhibitory cytokine 1
TGF-β	transforming growth factor-β
GFRAL	the glial cell line-derived neurotrophic factor receptor alpha-like
GAPDH	human glyceraldehyde 3-phosphate dehydrogenase
Ct	cycle threshold
RT-qPCR	reverse transcription quantitative polymerase chain reaction
ELISA	enzyme-linked immunosorbent assay
